# Molecular Rapid Diagnostics Improve Time to Effective Therapy and Survival in Patients with Vancomycin-Resistant *Enterococcus* Bloodstream Infections

**DOI:** 10.3390/antibiotics12020210

**Published:** 2023-01-19

**Authors:** Sarah M. Bandy, Christopher B. Jackson, Cody A. Black, William Godinez, Gerard W. Gawrys, Grace C. Lee

**Affiliations:** 1 College of Pharmacy, The University of Texas at Austin, Austin, TX 78712, USA; 2 Pharmacotherapy Education and Research Center, School of Medicine, UT Health San Antonio, 7703 Floyd Curl Drive, San Antonio, TX 78229, USA; 3 University Health System, 4502 Medical Drive, San Antonio, TX 78229, USA; 4 Methodist Hospital and Methodist Children’s Hospital, 7700 Floyd Curl Drive, San Antonio, TX 78229, USA

**Keywords:** rapid diagnostics, antibiotic resistance, bloodstream infection

## Abstract

Delays in appropriate antibiotic therapy are a key determinant for deleterious outcomes among patients with vancomycin-resistant Enterococcus (VRE) bloodstream infections (BSIs). This was a multi-center pre/post-implementation study, assessing the impact of a molecular rapid diagnostic test (Verigene^®^ GP-BC, Luminex Corporation, Northbrook, IL, USA) on outcomes of adult patients with VRE BSIs. The primary outcome was time to optimal therapy (TOT). Multivariable logistic and Cox proportional hazard regression models were used to determine the independent associations of post-implementation, TOT, early vs. delayed therapy, and mortality. A total of 104 patients with VRE BSIs were included: 50 and 54 in the pre- and post-implementation periods, respectively. The post- vs. pre-implementation group was associated with a 1.8-fold faster rate to optimized therapy (adjusted risk ratio, 1.841 [95% CI 1.234–2.746]), 6-fold higher likelihood to receive early effective therapy (<24 h, adjusted odds ratio, 6.031 [2.526–14.401]), and a 67% lower hazards for 30-day in-hospital mortality (adjusted hazard ratio, 0.322 [0.124–1.831]), after adjusting for age, sex, and severity scores. Inversely, delayed therapy was associated with a 10-fold higher risk of in-hospital mortality (aOR 10.488, [2.497–44.050]). Reduced TOT and in-hospital mortality were also observed in subgroups of immunosuppressed patients in post-implementation. These findings demonstrate that the addition of molecular rapid diagnostic tests (mRDT) to clinical microbiology and antimicrobial stewardship practices are associated with a clinically significant reduction in TOT, which is associated with lower mortality for patients with VRE BSIs, underscoring the importance of mRDTs in the management of VRE infections.

## 1. Introduction

Enterococcal blood-stream infections (BSIs) are associated with significant morbidity and mortality globally, with mortality rates ranging from 20% and 60% [1,2,3,4,5,6,7,8,9,10,11]. The treatment of enterococcal infections is increasingly challenging with the increased rates of vancomycin-resistant enterococci (VRE) reported from around the globe, including in the U.S., Europe, and Asia [1,2,5,11]. This is further compounded by the emergence of resistance to the few viable treatments for VRE BSIs, such as linezolid and daptomycin [8,9,10].

The U.S. Centers for Disease Control and Prevention (CDC) estimated that VRE caused over 54,500 infections among hospitalized patients and 5400 estimated deaths nationally since 2017 [2]. Compared to BSIs due to vancomycin-susceptible enterococcus, VRE BSIs have been associated with up to a 2.5-fold higher mortality [8,9,10,11,12]. Furthermore, VRE colonization and bacteremia are significant clinical complications that disproportionately impact immunocompromised hosts, including patients undergoing hematopoietic stem cell transplantation (HSCT) [12,13,14,15]. Notably, prior reports have demonstrated that delays in appropriate antibiotic therapy is a key determinant for deleterious outcomes due to VRE, underscoring the significant role molecular rapid diagnostic tests (mRDTs) may play to help in the management of VRE BSIs, including providing shorter time to optimal therapy (TOT) [16]. 

Compared to the 24–72 h turn-around time of conventional clinical microbiological processes, mRDTs, such as the Verigene^®^ Blood Culture Gram-Positive (BC-GP) panel (Luminex Corporation, Northbrook, IL, USA), have demonstrated high accuracy in the identification of Gram-positive pathogens, including *E. faecium* and *E. faecalis*, and detecting *vanA* and *vanB* genes within 1–2 h of positive Gram-stain [17]. In conjunction with antimicrobial stewardship programs (ASPs) and clinical microbiological practices, mRDTs have been shown to significantly reduce TOT by 6 to 43 h [18,19]. However, the impact of reduced TOT on outcomes for patients with BSIs, including survival, have been inconsistent across various Gram-positive and Gram-negative pathogens [16]. This is likely attributable to the interaction of factors related to prevalent host features (e.g., immunocompromised) and pathogen features (e.g., pathogenicity and drug-resistance profiles) where the greatest benefit has been shown for BSIs caused by resistant organisms in identified high-risk populations [16]. A prior study reported up to a 3-fold increase in 30-day mortality in the absence of effective therapy in the first 48 h for VRE BSIs [20]. Moreover, as VRE has a disproportionate deleterious impact on vulnerable populations (i.e., immunocompromised hosts) juxtaposed with the availability of highly effective antimicrobials against VRE that are infrequently used empirically, we surmised that the improvement of TOT for VRE BSIs are likely to yield a higher effect size on survival. However, limited studies have evaluated the proximate impact of the shorter TOT associated with mRDT on survival, particularly in VRE BSIs. 

Herein, we assess the impact of the mRDT (Verigene^®^ GP-BC) and ASPs on TOT and in-hospital mortality in patients with VRE BSIs within four community hospitals. This analysis also evaluated the differential impact of delayed TOT in early disease on outcomes in the overall cohort and in subgroups of high-risk patients (e.g., HSCT recipients and those with underlying hematological malignancies).

## 2. Results

This was a multi-center retrospective cohort study conducted at four community hospitals in San Antonio, Texas. In 2014, Verigene^®^ BC-GP mRDT and VigiLanz^®^ (VigiLanz Corporation, Minneapolis, MN, USA) surveillance software was implemented with the integration of real-time notifications to clinical pharmacists occurring in 2015. Inpatient adults with documented VRE BSIs between May 2011 and May 2013 (pre-implementation period) and between May 2015 and May 2017 (post-implementation period) were evaluated. The implementation and role of ASP pharmacists are detailed in a prior study [21]. Patients were included if they were aged 18 years or older, hospitalized, had a blood culture positive for VRE, and received at least one dose of optimized therapy, defined as an antibiotic regimen to which VRE was susceptible in vitro in accordance with the Clinical and Laboratory Standards Institute guidelines [22,23,24,25]. Only initial VRE BSI events were included from patients with multiple episodes [22]. Patients were excluded if they were not admitted to the hospital or were discharged or transferred before blood culture positivity. The study was approved by the Institutional Review Board of the Methodist Healthcare System with a waiver of informed consent.

### 2.1. Baseline Characteristics

**Figure 1:**
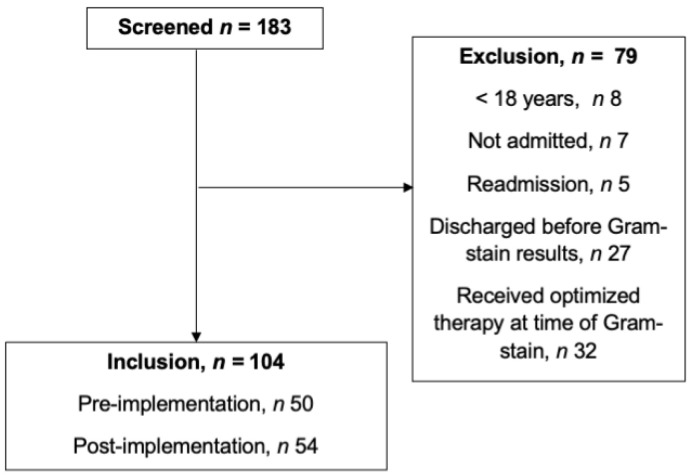
Cohort flow chart.

A total of 183 patients were identified with a VRE BSI during the study period (Figure 1). After screening, 104 patients were included in the study: 50 patients in the pre-implementation and 54 in the post-implementation group (Figure 1). 

There were no differences in baseline characteristics between the pre- and post-implementation groups (Table 1). The overall median age was 57 years (interquartile range (IQR), 46–69); 61% were males. The median Pitt bacteremia score (PBS) was 2 (IQR, 1–5) and 60% required admission to the intensive care unit (ICU). HSCT patients and those with neutropenia comprised 35% and 31% of the overall cohort, respectively; there were no differences in these conditions between implementation periods. Rates of VRE remained consistent throughout the duration of the study (range: 7.5–9%, Table S1); all enterococcal isolates tested against daptomycin or linezolid were susceptible according to Clinical and Laboratory Standards Institute (CLSI) breakpoints [25].

### 2.2. Implementation of mRDT ASP on Time to Optimal Therapy

**Figure 2:**
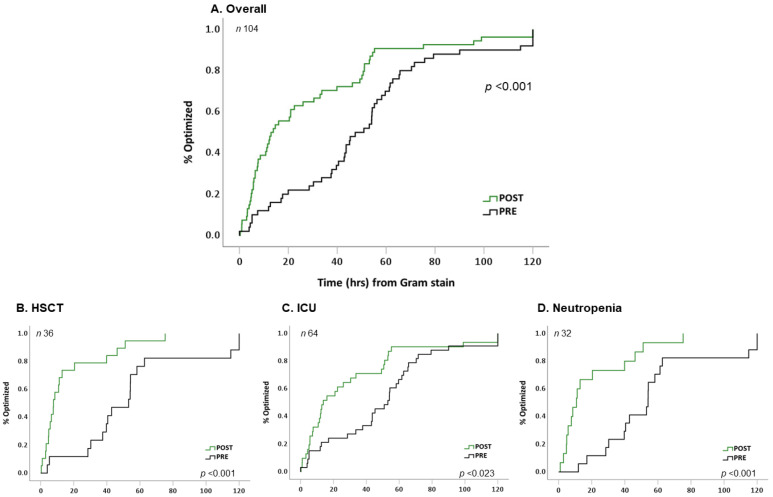
Time to optimal therapy by pre- or post-implementation. Kaplan–Meier (KM) plot depicting time to optimal therapy over 120 h from time of Gram-stain stratified by implementation group in the (**A**) overall cohort, (**B**) hematopoietic stem cell transplantation (HSCT) subgroup, (**C**) Intensive care unit (ICU) subgroup, (**D**) neutropenia subgroup. *p* by log-rank test. Black line, pre-implementation group; green line, post-implementation group.

The median TOT was nearly 4-fold shorter in the post-implementation group compared to the pre-implementation group (13 h [IQR, 6–50] vs. 49 h [IQR, 30–63], respectively; Table 2). Most patients in both groups were optimized to daptomycin (86%); there were no significant differences in selection of optimized agent or dosing (e.g., daptomycin, linezolid) between implementation periods (Table 2). After adjusting for age, sex, and PBS, the post-implementation group was associated with a 1.8-fold faster rate to TOT (adjusted risk ratio, 1.841 95% CI 1.234–2.746) than the pre-implementation group (Figure 2, Table 3). Moreover, 70% vs. 30% of patients in the post- vs. pre-implementation group, respectively, were placed on early optimized therapy (Table 2). 

The secondary subgroup analysis is described in Figure 2. When stratified by HSCT recipients, ICU level of care, and those with neutropenia, TOT was consistently shorter in the post-implementation period. In addition, there was no difference between median TOT between ASP staffing models during the post-implementation period (Table S2).

### 2.3. Implementation of Verigene^®^ BC-GP mRDT ASP on Mortality

Overall, all-cause in-hospital mortality occurred in 21% of the cohort. The post-implementation group had a significantly lower in-hospital mortality compared to the pre-implementation group (11% vs. 32%; Table 2). Baseline characteristics for in-hospital survivors vs. non-survivors are detailed in Table S3. A higher proportion of non-survivors had ICU admission and had higher PBS (Table S3). To determine the effect of covariates (age, sex, ICU, and PBS) and the relationship between the post-implementation and in-hospital mortality, we performed a series of logistic regression analyses, which are depicted in Table S4. Across all models, after adjusting for age, sex, and severity of illness (PBS and ICU admission), the post-implementation group remains independently associated with reduced in-hospital mortality (adjusted odds ratio, aOR 0.211, 95% CI 0.061–0.732; Table S4; model 7). Consistently, when stratified by HSCT recipients, ICU levels of care, and those with neutropenia, the post-implementation group was independently associated with a significantly lower risk for in-hospital mortality (Table S5). 

**Figure 3:**
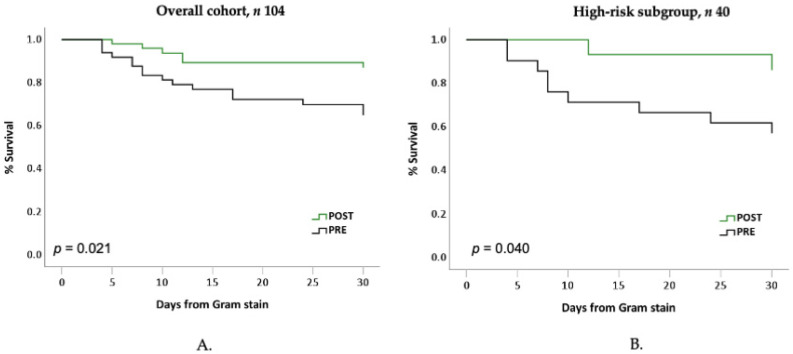
Adjusted odds ratio for in-hospital mortality during post-implementation vs. pre-implementation by subgroup. Kaplan–Meier (KM) plots depicting 30-day mortality hazards from time of Gram-stain stratified by implementation group in the (**A**) overall cohort and (**B**) high-risk subgroup comprising hematopoietic stem cell transplantation (HSCT), neutropenia (absolute neutrophil count [ANC] < 500 cells/mm^3^), active hematological malignancies, and solid organ transplant recipients. *p* by log-rank test. Black line, pre-implementation group; green line, post-implementation group.

The post-implementation group had significantly lower hazards for 30-day in-hospital mortality compared to the pre-implementation group (Figure 3A). In Cox proportional hazard models, the relationship between the post-implementation group and lower 30-day mortality persisted, independent of age, sex, and PBS (Table S6). Similarly, in the pooled high-risk subgroup of HSCT recipients, those with neutropenia and/or active malignancies, the post-implementation status consistently associated with a significantly lower hazard for 30-day in-hospital mortality (Figure 3B).

### 2.4. Early Time to Optimal Therapy and Mortality

The median TOT was 24 hours among survivors, which was half that observed compared to non-survivors (24.2 [7.1–51.6] vs. 54.1 [32.4–63.4], *p* <0.001; Table S3). Six percent of non-survivors received optimal therapy within early disease (within 24 hours) compared to 47% among survivors (*p* = 0.012). Inversely, those who received delayed optimal therapy (after 24 hours post-Gram stain) had greater than a 10-fold higher risk of mortality, after adjusting for age, sex, and PBS (aOR 10.488, 95% CI 2.497–44.050, Table S7).

## 3. Discussion

This study assessed the impact of implementing an mRDT (Verigene^®^ BC-GP) on VRE BSIs and determined the effects of early TOT on outcomes. Post-implementation, there was a significant reduction in TOT, as well as mortality. Furthermore, the results suggested that delayed TOT is associated with higher mortality. The shorter TOT and reduced in-hospital mortality post-implementation were also observed in high-risk subgroups of HSCT recipients, those with neutropenia and/or active malignancies. 

Multiple prior studies evaluating the impact of mRDTs in conjunction with ASPs have found significantly lower TOT, while others have demonstrated increased deleterious outcomes with delays in timely appropriate therapy [16,20,26]. Herein, we show that the longer TOT in the pre-implementation group significantly delayed time to optimal therapy, which directly increased the risk of mortality by 10-fold. This finding contrasts with a previous study assessing Verigene BC-GP for enterococcal bacteremia, which found no mortality differences between pre- and post-implementation groups [23]. Differences could be due to the inherent cohort differences, length of time during implementation periods, and/or that both vancomycin susceptible and non-susceptible Enterococcus spp. were assessed. Notably, the cohort in our study comprised of a high proportion of immunocompromised patients, namely HSCT recipients and those with active malignancies, groups that have been previously established to be more likely to experience poor outcomes due to delayed treatment in VRE BSIs [13,14,15,27,28,29,30,31]. We performed a series of stratified and subgroup analyses and found consistent relationships between shorter TOT, post-implementation, and in-hospital mortality. These findings are not as surprising as a number of factors that place patients undergoing HSCT at higher risk for serious VRE infections (e.g., neutropenia, prolonged and repeated healthcare and antibiotic exposures, and widespread use of central venous catheters) [31]. Thus, it is not surprising that profound benefits of shorter TOT were observed in this population, where VRE has become the leading cause of BSIs [31]. Moreover, we estimate that mRDTs in conjunction with ASP would need to be utilized in approximately five patients with VRE BSI to prevent one in-hospital death, providing support to mRDTs as part of the standard of care, particularly in institutions serving high-risk immunocompromised patients.

These associations of TOT and mortality parallels the findings of a meta-analysis of 31 studies wherein mRDTs were found to have improved TOT, as well as mortality benefits, compared to conventional microbiological procedures for both Gram-positive and Gram-negative organisms [16]. For example, lower mortality was observed in Staphylococcus spp. BSIs after the implementation of a peptide nucleic acid fluorescence in situ hybridization (PNA FISH) mRDT while another study identified a nearly 50% reduction in 30-day all-cause mortality from Gram-negative BSIs [16,32]. 

Between the cohorts, there was no difference in the selection of optimized therapy for the treatment of VRE BSIs. The majority of patients received an empiric regimen comprising of vancomycin. Among survivors, the median TOT was 24 h after Gram-stain; other studies (without mRDTs) have suggested for optimal therapy within 48 h of disease onset [20]. However, a major chasm in clinical practice is that organism susceptibility results from standard microbiological procedures requires 48–72 h, underscoring the critical role of mRDTs in the management of optimizing outcomes among those with VRE BSIs. Various mRDT platforms, such as the one implemented in this study (Verigene^®^) have the capability to detect Enterococcal species, as well as vanA and vanB genes, within a couple hours of positive blood culture, thereby allowing practitioners to strive for early optimal therapy, which is critical for survival in patients with VRE BSIs [17]. A meta-analysis of mRDTs in a VRE BSI subgroup found improvements in time to effective therapy by >24 h [16]. Furthermore, this study found no difference in TOT between ASP staffing models (full vs. partial), indicating the consistency of ASP processes, as well as the high uptake of pharmacists’ recommendations among providers. 

This study has limitations. First, this was a retrospective study design with inherent potential for uncontrolled confounders and bias. Potential prescriber bias was limited by the pre-implementation and post-implementation study design in which all patients within each group were observed over the same period of time, times of year, and under the same conditions (pharmacy staffing, ASP implementation, rates of VRE BSI, and conventional microbiological methods), with the exception of the implementation of Verigene^®^ BC-GP and Vigilanz^®^ clinical surveillance software. Furthermore, this study included 24 months of data per cohort, allowing adequate time to evaluate TOT in VRE BSIs despite inherent provider variability. Additionally, a conservative time for implementation of Verigene^®^ BC-GP and Vigilanz^®^ clinical surveillance software was permitted between cohorts. This implementation period ensured clinicians were familiar with the mRDT combined with the ASP prior to the post-implementation period. Moreover, there were no differences observed in baseline characteristics between groups. Second, clinical resolution and microbiologic cure were not assessed. Third, the majority (80%) of patients were optimized to daptomycin, with most patients managed on standard dosing. This was not a surprising finding, considering a significant portion of the study cohort comprised of HSCT or neutropenic patients who may not tolerate the potential hematologic adverse effects of linezolid (e.g., myelosuppression) for an extended period [33]. Thus, these findings may not be generalizable to institutions that favor linezolid as their agent of choice for VRE BSIs. However, in subgroup analyses, lower TOT and mortality were observed in patients with and without neutropenia. Additionally, at the time the pre- and post-implementation period were commencing, limited data were available comparing standard to high-dosing daptomycin for VRE BSI, which may explain the higher proportion of patients managed on standard dosing. Lastly, despite significantly reduced TOT in the post-implementation cohort, this study did not find a significant difference in either hospital or ICU length of stay. This is consistent with other studies that have shown mixed results regarding significant reductions in LOS after the implementation of mRDTs (22,26–30), which may be attributable to the higher comorbid burden and the complexity of the study population impacting LOS.

In conclusion, the addition of an mRDT tool to conventional pathogen identification and ASP was associated with significantly reduced TOT and all-cause in-hospital mortality for patients with VRE BSIs. These findings highlight the importance of strategies, including mRDTs for early optimization of therapy for patients with VRE infections, particularly among high-risk groups (HSCT and neutropenic patients), where delayed effective antimicrobial therapy can have detrimental implications.

## 4. Materials and Methods

This was a multi-center study assessing the impact of a mRDT (Verigene^®^ GP-BC, Luminex Corporation, Northbrook, IL, USA) for the detection of VRE in adults patients with BSIs. Patients were compared between two different 24-month periods; the pre-implementation period consisting of conventional microbiological methods and the post-implementation period consisting of Verigene^®^ GP-BC, in addition to conventional microbiological methods. There were 12 months between periods for implementation and training on Verigene^®^ GP-BC. 

### 4.1. Implementation Phases

#### 4.1.1. Pre-Implementation

During this period, blood culture specimens were obtained using conventional methodology and incubated in a BACT/ALERT^®^ (bioMérieux, Marcy l’Etoile, Auvergne, France) system. Upon positivity, a Gram-stain was performed, and the results of the Gram-stain were communicated via telephone to providers and recorded through this facility’s electronic health record (EHR). Identification and susceptibility were conducted using VITEK 2 Gram-positive ID cards (bioMérieux, Marcy l’Etoile, Auvergne, France) and expert panel software that were released into the EHR.

#### 4.1.2. Implementation

In August 2014, the use of VigiLanz^®^ (VigiLanz Corporation, Minneapolis, MN, USA) clinical surveillance software and Verigene^®^ BC-GP test were both implemented. Verigene^®^ BC-GP mRDT was performed 24 h a day, 7 days a week in the four community hospitals. No mRDTs were available prior to the implementation of the Verigene^®^ BC-GP mRDT. VigiLanz^®^ is a clinical surveillance software that combines data from multiple sources, including Verigene^®^, and is independent of the EHR [17,21].

#### 4.1.3. Post-Implementation

Verigene^®^ BC-GP mRDT and real-time VigiLanz^®^ notifications were added onto the pre-implementation methods. No changes to blood culture methods or susceptibility reporting were implemented during either study period. VRE was identified using Verigene^®^ BC-GP mRDT through the detection of resistance markers (*vanA* or *vanB*) within two to four hours of positive blood cultures and confirmed with phenotypic results once available. Clinical pharmacists review and communicate the mRDT results to providers. Provider education was provided for the duration of the post-implementation period. 

### 4.2. Data Collection and Definitions

Data collected from the EHRs included patient demographics, microbiological data, antimicrobial data, and clinical outcomes. PBS was calculated to assess the severity of patient illness [22,23]. Patients were followed through their entire hospital course until discharge or expiration. 

VRE BSIs were defined as BSIs due to an *Enterococcus* spp. resistant to vancomycin phenotypically and/or genotypically through the detection of the *vanA*/*vanB* genotype. Genotypic results were confirmed phenotypically through susceptibility testing. Empiric antibiotic therapy was defined as therapy employed prior to release of antibiotic susceptibility results, whereas definitive therapy was defined as therapy given after release of antibiotic susceptibility results [22]. Optimization potential was defined as a patient with a VRE BSI that required changes/escalation of antimicrobial therapy based on phenotypic and/or genotypic results. Optimal therapy was defined as an antibiotic regimen to which the VRE was susceptible in vitro in accordance with the Clinical and Laboratory Standards Institute guidelines [25]. Daptomycin, linezolid, and penicillins based on phenotypic and/or genotypic results were considered optimized therapies. For the purposes of this study, daptomycin dosing was considered ‘standard dose’ if 4–6 mg/kg and ‘high dose’ if greater than 6 mg/kg. TOT was defined in hours as the difference between the recorded time of Gram-stain results and the initiation of optimized antimicrobial therapy. ASP staffing models are reported as full-staffed and partial-staffed. An infectious disease pharmacist along with clinical pharmacists in a decentralized pharmacy model served as the full-staffed ASP vs. decentralized pharmacy, which was only considered partial-staffed. Full-staffed ASP times were weekdays (Monday–Friday) during the day/evening shift (0700 to 2000). Partial-staffed ASP times were considered outside this timeframe when fewer pharmacists were available at the included hospitals. 

### 4.3. Outcomes

The primary outcome of this study was to evaluate TOT in patients with VRE BSIs before and after our implementation period. Secondary outcomes included in-hospital mortality, median duration of hospital and ICU stay, and TOT within 24 h. In-hospital all-cause 30-day mortality occurred if an admitted patient expired during their index hospitalization. Hospital length of stay (LOS) was defined as the difference in days between hospital admission and hospital discharge or expiration. ICU LOS was defined as the difference in days between admission and discharge from or expiration in the ICU. Subgroup analysis focused on high-risk patients, including HSCT recipients, neutropenia (absolute neutrophil count [ANC] < 500 cells/mm^3^), active hematological malignancies, and solid organ transplant recipients.

### 4.4. Statistical Analyses

Demographic and clinical variables were expressed as n (%) for categorical variables and median (IQR) for continuous variables. Bivariable comparisons of characteristics and outcomes between pre- and post-implementation groups were conducted using χ^2^ tests or Fisher exact tests, as well as Student’s t tests and Mann–Whitney U tests, as appropriate. The primary outcome of TOT was analyzed for all included patients, as well as subgroups. With an average TOT of 24 h, to detect a 50% difference, an α-value of 0.05, and a power of 80% it was determined a priori that a minimum of 44 patients would be needed in each group. 

First, we described the cohort characteristics for the primary outcome of TOT by implementation group (pre- vs. post-implementation) and separately for patients who died in hospital (hospital non-survivors) and hospital survivors. Following bivariate analyses, multivariable logistic regression was conducted to assess independent association between the implementation period with TOT and mortality, as well as other clinical conditions. Variables that differed between groups in the bivariable analysis (indicated with *p* < 0.2) were included in the multivariable models, in addition to established clinically relevant variable, such as age and severity metric (PBS) [19,20]. All multivariable models computed included the implementation period (pre vs. post) adjusted by age, sex, and PBS. Full model iterations are included in the supplement showing step-wise adjustments for each variable (age, sex, PBS, and high-risk patient factors (i.e., neutropenia, malignancies, and ICU admission)) to assess the independent association of the implementation period on in-hospital mortality. In addition, models for in-hospital mortality were conducted to assess the differential impact of TOT during early disease (24 h post-Gram stain). TOT and in-hospital survival was investigated using Kaplan–Meier plots and log-rank test for comparison. Cox proportional hazards models were used to estimate the association between post-implementation with TOT and 30-day in-hospital mortality. Statistical significance was calculated using a *p*-value of <0.05. Adjusted odds ratios (aOR), hazard ratios (aHR), or risk ratios (aRR), as well as their 95% confidence intervals (CIs), are reported. SPSS 28.0^®^ (IBM Crop, Armonk, NY, USA) was used for all statistical analyses. 

## Figures and Tables

**Table 1:** Baseline characteristics.

Characteristic	Pre-Implementation (*n* = 50)	Post-Implementation (*n* = 54)	*p*
Age (years), median (IQR)	58 (46.8–72.0)	57 (42.5–68.0)	0.411
Sex (male), n (%)	27 (54)	36 (66.7)	0.187
ICU admission, n (%)	33 (66)	31 (57.4)	0.368
Pitt bacteremia score (IQR)	2.0 (0–5)	2.0 (0–4)	0.436
High-risk patients *, n (%)	24 (48)	28 (51.8)	0.971
HSCT recipient	17 (34)	19 (35.2)	0.899
Active malignancies	4 (8)	5 (9.3)	1.000
Solid organ transplant	3 (6)	4 (7.4)	1.000
Neutropenia (ANC < 500 cells/mm^3^), n (%)	17 (34)	15 (27.8)	0.492
Infection sources, n (%)	-	-	0.893
Gastrointestinal/intra-abdominal	31 (62)	32 (59.3)	-
Catheter-related	6 (12)	10 (18.5)	-
Skin and skin structure	4 (8)	4 (7.4)	-
Urinary/genitourinary	5 (10)	6 (11.1)	-
Unknown	4 (8)	2 (3.7)	-

* Patients may have more than one high-risk condition. IQR: interquartile range; ICU: intensive care unit; ANC: absolute neutrophil count; HSCT: hematopoietic stem cell transplant.

**Table 2:** Outcomes by implementation group.

	Pre-Implementation (n = 50)	Post-Implementation (n = 54)	*p*
Median (IQR) time (hours) from GS to antimicrobial therapy optimization	49.1 (29.9–63.4)	13.4 (5.7–49.5)	0.034
VRE coverage at 24 h from GS, n (%)	15 (30.0)	38 (70.4)	<0.001
Optimized antibiotic, n (%)			0.356
Linezolid	8 (16.0)	4 (7.4)	-
Daptomycin ^†^	41 (82.0)	48 (88.9)	0.244
Standard dose	28 (68.3)	27 (56.3)	-
High dose	13 (31.7)	21 (43.8)	-
Piperacillin- tazobactam ^§^	1 (2.0)	2 (3.7)	-
In-hospital all-cause mortality, n (%)	16 (32.0)	6 (11.1)	0.009
Median (IQR) hospital LOS (days) *	19.8 (12.2–47.5)	23.5 (14.3–39.2)	0.959
Median (IQR) ICU LOS (days) *	4.1 (2.8–6.8)	6.0 (2.4–15.3)	0.775

GS, Gram-stain; VRE, vancomycin-resistant *Enterococcus* spp.; ICU, intensive care unit; LOS, length of stay; ^†^ High dose defined as >6 mg/kg; ^§^ Susceptibility confirmed through standard procedures using Vitek ^®^ 2 (bioMérieux Inc., Marcy l’Etoile, Auvergne, France); * Length of stay estimates are among in-hospital survivors.

**Table 3:** Cox proportional model for time to optimal therapy.

	aRR (95% CI)	*p*
Post-implementation group	1.841 (1.234–2.746)	0.003
Age	0.996 (0.984–1.007)	0.459
Sex	0.936 (0.626–1.401)	0.749
PBS	1.000 (0.920–1.087)	0.996

aRR, adjusted rate ratio; TOT, time to optimal therapy; PBS, Pitt bacteremia score. Cox proportional hazards models were used to estimate the association between rates to optimal therapy of post-implementation with TOT.

## Data Availability

Data supporting results will be shared upon request.

## Supplementary Materials

The following supporting information can be downloaded at: https://www.mdpi.com/article/10.3390/antibiotics12020210/s1, Table S1: VRE Rates from 2013–2017; Table S2: ASP Staffing on Time to Optimal Therapy by Implementation Group; Table S3: Demographics and Baseline Characteristics of Survivors vs. Non-survivors; Table S4: Multivariable Models for In-Hospital All-Cause Mortality in Overall Cohort; Table S5: Models for In-Hospital All-Cause Mortality by Subgroups; Table S6: Cox Proportional Hazard for 30-day In-Hospital All-Cause Mortality; Table S7: Multivariable Models for In-Hospital All-Cause Mortality by Delayed Optimal Therapy.
