# Supplementary material for: Molecular Rapid Diagnostics Improve Time to Effective Therapy and Survival in Patients with Vancomycin-Resistant Enterococcus Bloodstream Infections
Source: Antibiotics (Basel). 2023 Jan 19;12(2):210. doi: 10.3390/antibiotics12020210 (PMC9952532; doi:10.3390/antibiotics12020210)
Supplement: Supplementary file 1 [file antibiotics-12-00210-s001.zip › antibiotics-2145631-supplementary.pdf]

Table S1. VRE Rates from 2013 to 2017

Year	VRE rates per 1,000 Enterococcal spp. isolates
12/31/2016-12/31/2017	75.1
12/31/2015-12/31/2016	90.9
12/31/2014-12/31/2015	83.5
12/31/2013-12/31/2014	96.6

VRE: vancomycin resistant *Enterococcus* spp. Rates are computed per 1,000 *Enterococcus* spp. isolates within the four community hospitals over each respective year. *Enterococcus gallinarum* was excluded.

Table S2. ASP Staffing on Time to Optimal Therapy by Implementation Group

	**Fully Staffed**	**Not Fully Staffed**	***P***
Pre-Implementation, median (IQR) TOT (n=50)	43.7 (10.1-62.8)	54.1 (38.6-64.0)	0.190
Post-Implementation, median (IQR) TOT (n=54)	20.1 (5.7-50.2)	12.3 (5.4-32.4)	0.332

ASP: Antimicrobial stewardship program; IQR: interquartile range; TOT: time to optimal therapy

Table S3. Demographics and Baseline Characteristics of Survivors vs. Non-Survivors

Characteristic	Survivors	Non-Survivors	*P*
Age (years), median (IQR)	57.0 (44.8-82.7)	54.5 (46.8-74.0)	0.650
Sex (male), n (%)	47 (57.3)	16 (72.7)	0.189
ICU admission, n (%)	44 (53.7)	20 (90.9)	0.001
PBS, median (IQR)	1.0 (0-3.25)	5.0 (2.75-6.0)	<0.001
High-risk, n (%)			
HSCT recipient	27 (32.9)	9 (40.9)	0.485
Active malignancies	7 (8.5)	2 (9.1)	0.935
Solid organ transplant	6 (7.3)	1 (4.5)	0.645
Neutropenia (ANC < 500 cells/mm^3)	22 (26.8)	10 (45.5)	0.120
Infection sources, n (%)			0.302
Gastrointestinal/intra-abdominal	49 (59.8)	14 (63.6)	
Catheter-related	11 (13.4)	5 (22.7)	
Skin and skin structure	6 (7.3)	2 (9.1)	
Urinary/genitourinary	10 (12.2)	1 (4.5)	
Unknown	6 (7.3)	0 (0)	
**Outcomes**			
Median (IQR) time (hours) from GS to antimicrobial therapy optimization	24.2 (7.1-51.6)	54.1 (32.4-63.4)	<0.001
VRE Coverage at 24 hours from GS, n (%)	47 (57.3)	6 (27.3)	0.012

IQR: Interquartile range; ICU: intensive care unit; ANC: absolute neutrophil count. Infection sources compared using Fishers Exact test.

Table S4. Multivariable Models for In-Hospital All-Cause Mortality in Overall Cohort

	aOR (95% CI)	*P*
**Model 1**		
TOT	1.001 (0.996-1.005)	0.708
**Model 2**		
Post-Implementation	0.254 (0.089-0.724)	0.010
Age	0.988 (0.961-1.015)	0.384
**Model 3**		
Post-Implementation	0.221 (0.075-0.647)	0.006
Age	0.988 (0.960-1.017)	0.412
Sex	0.398 (0.134-1.186)	0.098
**Model 4**		
Post-Implementation	0.219 (0.064-0.742)	0.015
Age	0.977 (0.943-1.012)	0.194
Sex	0.324 (0.090-1.170)	0.085
PBS	1.573 (1.243-1.989)	<0.001
**Model 5**		
Post-Implementation	0.233 (0.064-0.842)	0.026
Age	0.974 (0.937-1.012)	0.180
Sex	0.281 (0.072-1.100)	0.068
PBS	1.765 (1.326-2.346)	<0.001
Neutropenic	5.786 (1.440-23.244)	0.013
**Model 6**		
Post-Implementation	0.211 (0.060-0.741)	0.015
Age	0.981 (0.945-1.018)	0.298
Sex	0.319 (0.085-1.199)	0.091
PBS	1.682 (1.291-2.193)	<0.001
HSCT/Active malignancy	2.787 (0.767-10.119)	0.119
**Model 7**		
Post-Implementation	0.211 (0.061-0.732)	0.014
Age	0.301 (0.083-1.101)	0.070
Sex	0.980 (0.945-1.015	0.259
PBS	1.433 (1.106-1.858)	0.007
ICU admission	0.288 (0.049-1.676)	0.166

aOR: Adjusted odds ratio; CI: confidence interval; TOT: time to optimal therapy; PBS: Pitt bacteremia score; ICU: intensive care unit

Table S5. Models for In-Hospital All-Cause Mortality by Subgroups

	**aOR (95% CI)**	***P***
**Active Malignancy/Transplant, n=52**		
Post-implementation	0.116 (0.014-0.952)	0.045
Age	0.997 (0.920-1.038)	0.071
Female	0.241 (0.028-2.037)	0.140
PBS	1.879 (1.240-2.849)	0.002
**HSCT, n=36**		
Post-implementation	0.062 (0.004-0.880)	0.040
Age	0.943 (0.867-1.024)	0.164
Sex	0.301 (0.024-3.737)	0.350
PBS	3.478 (1.095-3.320)	0.023
**ICU Admission, n=64**		
Post-implementation	0.151 (0.037-0.620)	0.009
Age	0.994 (0.955-1.035)	0.778
Sex	0.194 (0.044-0.846)	0.029
PBS	1.393 (1.058-1.835)	0.018
**Neutropenic, n=32**		
Post-implementation	0.070 (0.000-0.827)	0.041
Age	0.870 (0.748-1.012)	0.071
Sex	0.065 (0.002-2.456)	0.140
PBS	3.478 (1.200-10.084)	0.022

aOR: Adjusted odds ratio; CI: confidence interval; TOT: time to optimal therapy; PBS: Pitt bacteremia score; ICU: intensive care unit. Models computed using logistic regression

Table S6. Cox Proportional Hazard Models for 30-day In-Hospital All-Cause Mortality

	**HR (95% CI)**	***P***
**Model 1**		
Post-implementation	0.318 (0.124-0.814)	0.017
Age	0.996 (0.973-1.018)	0.697
**Model 2**		
Post-implementation	0.292 (0.114-0.749)	0.010
Age	0.995 (0.972-1.018)	0.664
Sex	0.452 (0.176-1.163)	0.100
**Model 3**		
Post-implementation	0.322 (0.124-1.831)	0.019
Age	0.996 (0.968-1.024)	0.759
Sex	0.472 (0.182-1.221)	0.122
PBS< 3	6.669 (2.450-18.309)	<0.001

HR: Hazard ratio; CI: confidence interval; TOT: time to optimal therapy; PBS: Pitt bacteremia score

Table S7. Multivariable Models for In-Hospital All-Cause Mortality by Delayed Optimal Therapy

	**aOR (95% CI)**	***P***
No early optimal therapy	10.488 (2.497-44.050)	<0.001
Age	0.990 (0.932-1.009)	0.970
Female	0.266 (0.068-1.038)	0.057
PBS	1.806 (1.366-2.388)	<0.001

aOR: Adjusted odds ratio; CI: confidence interval; PBS: Pitt bacteremia score; ICU: intensive care unit. Models computed using logistic regression. Early optimal status classified as time to optimal therapy from Gram-stain of less than 24 hours.
